# New York

**Published:** 1897-02

**Authors:** 


*New York.*—*Assembly Bill No.* 231.—An Act in relation to the appointment of a State Veterinarian, and defining his duties. The people of the State of New York, represented in Senate and Assembly, do enact as follows:

SECTION 1. The Governor shall, with the advice and consent of the Senate, within ten days of the passage of this Act, appoint a State Veterinarian, who shall hold office for the term of three years; he shall be a regularly graduated veterinary surgeon of not less than ten years' continuous practice in this State, and shall be skilled in veterinary science; he shall be a member of the State Board of Health, which membership shall be in addition to that now provided by law; he shall receive from the State Treasurer as his compensation the sum of three hundred dollars per month and his actual expenses incurred while in the discharge of his duties, which shall be presented under oath and covered by written vouchers before receiving the same.

SEC. 2. It shall be the duty of the State Veterinarian to investigate any and all cases of contagious or infectious diseases among domestic animals of the State which may come to his knowledge or be brought to the notice of the State Board of Health or Commissioner of Agriculture, and for this purpose shall visit at once any locality within the State where any such contagious or infectious disease of domestic animals may be reported to exist, and make a full and careful examination of all animals supposed to be diseased, and to inquire into the nature and cause of any such disease which he may discover, prescribe the proper care, necessary remedies, and direct the sanitary measures necessary to prevent the spread thereof; to see that all State laws relating to infectious and contagious diseases of animals are enforced, and to make a full report of his findings and doings in the premises, with recommendations and suggestions as to the means necessary to be employed to prevent the spread, and best calculated to exterminate any and all such contagious or infectious diseases by him found to exist among the domestic animals of the State. All cases of tuberculosis in animals, or any infectious or contagious diseases of domesticated animals that may be brought to the notice of the State Board of Health or Commissioner of Agriculture, shall be referred to him, and he shall be placed in charge of any work that is undertaken by either the State Board of Health or Commissioner of Agriculture to eradicate tuberculosis or any infectious or contagious diseases of any animals, and make a full report of the same to the department which has placed him in charge thereof.

SEC. 3. All Acts or parts of Acts in conflict with any of the provisons of this Act are hereby repealed.

SEC. 4. This Act shall take effect immediately.

